# Programmed DNA elimination in the parasitic nematode *Ascaris*

**DOI:** 10.1371/journal.ppat.1011087

**Published:** 2023-02-02

**Authors:** Brandon Estrem, Jianbin Wang

**Affiliations:** 1 Department of Biochemistry and Cellular and Molecular Biology, University of Tennessee, Knoxville, Tennessee, United States of America; 2 UT-ORNL Graduate School of Genome Science and Technology, University of Tennessee, Knoxville, Tennessee, United States of America; Joan and Sanford I Weill Medical College of Cornell University, UNITED STATES

## Abstract

In most organisms, the whole genome is maintained throughout the life span. However, exceptions occur in some species where the genome is reduced during development through a process known as programmed DNA elimination (PDE). In the human and pig parasite *Ascaris*, PDE occurs during the 4 to 16 cell stages of embryogenesis, when germline chromosomes are fragmented and specific DNA sequences are reproducibly lost in all somatic cells. PDE was identified in *Ascaris* over 120 years ago, but little was known about its molecular details until recently. Genome sequencing revealed that approximately 1,000 germline-expressed genes are eliminated in *Ascaris*, suggesting PDE is a gene silencing mechanism. All germline chromosome ends are removed and remodeled during PDE. In addition, PDE increases the number of chromosomes in the somatic genome by splitting many germline chromosomes. Comparative genomics indicates that these germline chromosomes arose from fusion events. PDE separates these chromosomes at the fusion sites. These observations indicate that PDE plays a role in chromosome karyotype and evolution. Furthermore, comparative analysis of PDE in other parasitic and free-living nematodes illustrates conserved features of PDE, suggesting it has important biological significance. We summarize what is known about PDE in *Ascaris* and its relatives. We also discuss other potential functions, mechanisms, and the evolution of PDE in these parasites of humans and animals of veterinary importance.

## Introduction

*Ascaris* is a parasitic nematode that lives in the small intestine of humans and pigs [1]. The human (*A*. *lumbricoides*) and pig (*A*. *suum*) parasites can cross-infect and have little difference in anatomy, physiology, and genome sequences [2–6]. For simplicity, we use *Ascaris* to refer to both species in this review. *Ascaris* was estimated to infect >700 million people worldwide in 2021 [7]. Most cases are found in impoverished children [8–10]. *Ascaris* infections (ascariasis) often have mild or no symptoms, but heavy infections can cause intestinal blockages and even death if left untreated [8,10]. Ascariasis contributes significantly to global disability-adjusted life years and perpetuates the cycle of poverty [9,11].

*Ascaris* is a member of the Ascarididae parasitic nematode family, known as ascarids, that infect a wide variety of vertebrates, including *Parascaris univalens* and *P*. *equorum* (horse), *Toxocara canis* (dog) and *T*. *catti* (cat), *Baylisascaris procyonis* (raccoon) and *B*. *schroederi* (panda), as well as *Ascaridia galli* (chicken). Deworming drugs are widely used to attempt to control Ascarididae and other parasitic infections [3,8,12]. However, recurrent drug treatment has led to drug resistance in nematodes of veterinary importance [8,12–14]. Thus, new drugs are needed to fight the increasing drug resistance.

Ascarids have been used as models for both basic and biomedical research [1]. In addition to studies on the pathogenesis of *Ascaris* [15,16], ascarids have contributed to our understanding of meiosis [17,18], the chromosome theory of heredity, centrosome function in chromosome segregation [19,20], trans-splicing [21–24], neurobiology [25,26], metabolism [27–30], amoeboid sperm in nematodes [31–34], and programmed DNA elimination (PDE) [35–41]. This review will focus on *Ascaris* as a model for PDE, a form of genome alteration in which organisms reproducibly discard certain DNA sequences during early development. We summarize the current understanding of the PDE process in *Ascaris*, including the formation of DNA breaks, loss of sequences, and the potential molecular mechanisms, functions, and evolution of PDE in nematodes. We also discuss future directions and how PDE research can contribute to understanding the basic biology of these parasitic nematodes.

### Overview of PDE

**Fig 1:**
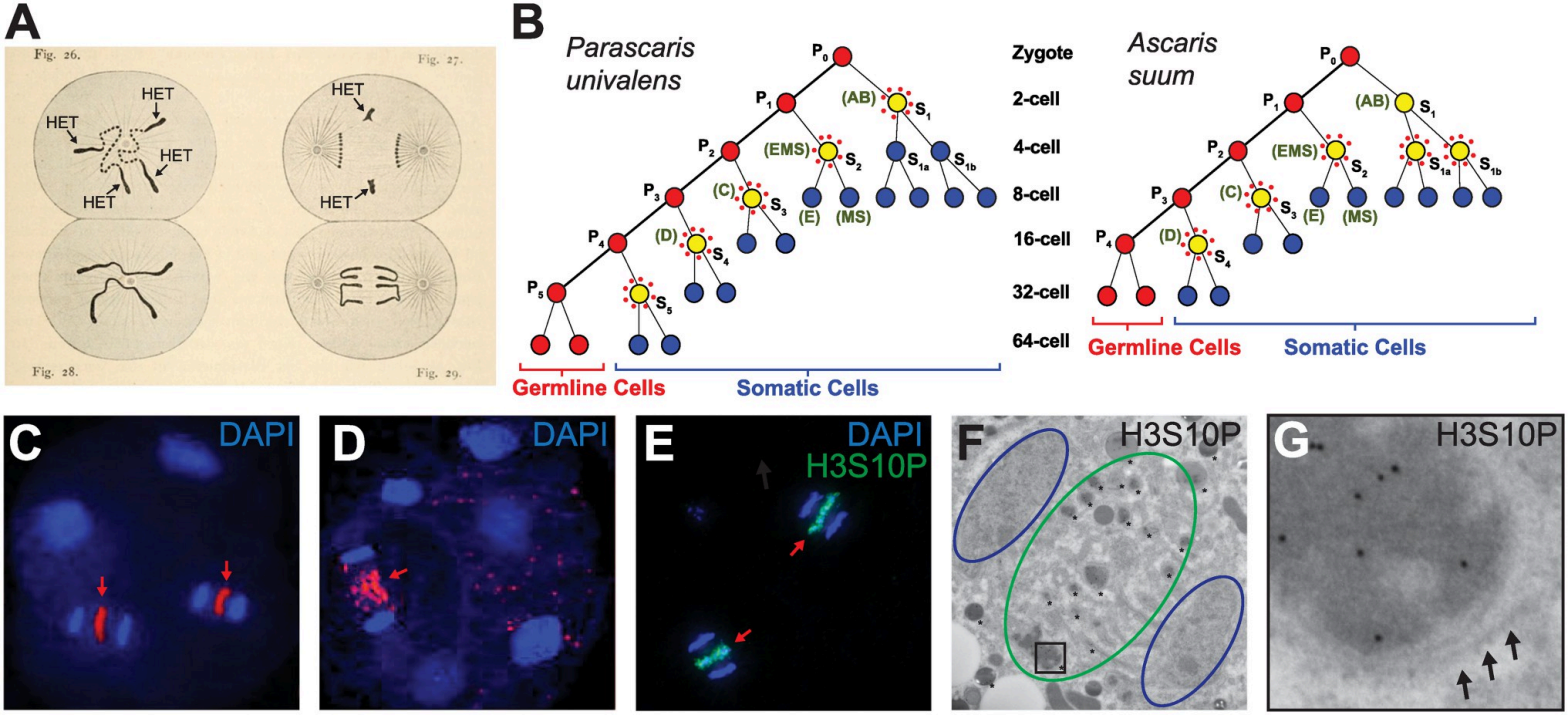
PDE occurs during nematode early embryogenesis. (**A**) Theodor Boveri’s drawing of *P*. *univalens* DNA elimination in a two-cell embryo. Left: interphase, chromosomes in the pre-somatic cell (top) are fragmented from PDE, while the chromosomes from the germ cell (bottom) are intact. Right: anaphase, the pre-somatic cell sequesters the chromosome fragments and leaves the HET arms at the metaphase plate. Image from Wikimedia commons. (**B**) *P*. *univalens* and *Ascaris* early cell lineage during early development. Germ cells are red, pre-somatic cells are yellow and surrounded by red dots, and somatic cells are blue. PDE occurs during successive divisions in early embryogenesis. Figure adapted from [37,38]. (**C**) A 4-cell *Ascaris* embryo with 2 cells undergoing PDE. DAPI staining showing eliminated DNA (artificially colored in red for emphasis) is left at the metaphase plate (red arrow) but retained DNA is segregated to the daughter nuclei. (**D**) A 6-cell *Ascaris* embryo with 1 cell undergoing PDE. Eliminated DNA begins to degrade during late anaphase (red arrow). Eliminated DNA fragments from previous PDE events are seen scattered across the embryo. Images in (**C**) and (**D**) are from [36]. (**E)** DAPI and H3S10P immuno-staining of *Ascaris* PDE. Eliminated DNA at the metaphase plate (red arrow) is stained with an antibody against H3S10P. (**F**) H3S10P Immuno-EM shows double-membrane bound H3S10P-positive structures between daughter nuclei during telophase of *Ascaris* PDE. Daughter nuclei are circled in blue. The region containing eliminated DNA is circled in green (for comparison with (**E**)). Double-membrane structures positive for H3S10P are marked with an asterisk (*). (**G**) Inset from (**F**). Arrows point to double-membrane structure. (**E**) and (**F**) are adapted from [41].

PDE was discovered in 1887 by cell biologist Theodor Boveri in the horse parasite *P*. *univalens* [42]. The *P*. *univalens* germline contains a single, large chromosome with 2 heterochromatic (HET) chromosome arms that are mainly composed of repeats (see below on the nature of eliminated DNA). Boveri observed that this single chromosome was fragmented during early embryogenesis in pre-somatic cells (Fig 1A). The resulting chromosome fragments were passed to the daughter nuclei, while the HET arms were eliminated from the reduced somatic genome [42] (Fig 1B). Since the discovery of PDE, single-cell ciliates and over 100 metazoan species from phylogenetically diverse groups, including nematodes, insects, mites, copepods, ratfishes, hagfishes, lamprey, songbirds, and bandicoots, have been observed to eliminate DNA [36,43]. However, within each phylogenetic group of metazoans, only a small percentage of organisms undergo PDE, suggesting PDE evolved independently among these groups [36,44].

Not surprisingly, the PDE processes are mechanistically diverse. In metazoans, there are 2 major types of PDE: (1) loss of an entire chromosome(s); and (2) fragmentation of chromosomes resulting in the loss of genetic material, coincident with germline-to-soma differentiation. The second form of PDE is particularly intriguing as it requires the generation of double-stranded DNA breaks (DSBs). Nematodes and ciliates are known to introduce DSBs during PDE [45]; based on cytological studies, other animals such as hagfish and copepods also likely break their chromosomes during PDE [46,47]. In single-cell ciliates, domesticated transposases are responsible for many of the PDE DNA breaks [48]. However, the mechanisms and machinery associated with the DNA breaks in metazoans remain largely unknown. More general descriptions and discussions on PDE in diverse organisms have been summarized in recent reviews [40,43,45,49–52].

*Ascaris* remains one of the most extensively studied models for PDE in metazoans. Early work in *Ascaris* provided robust cytological and molecular data that revealed critical features of *Ascaris* PDE [37,44,53]. Recent genomics studies have expanded our understanding of *Ascaris* PDE to the whole genome at the chromosomal level [41]. These studies are in part facilitated by the large size of *Ascaris* and its enormous reproductive capacity [1] with large numbers of zygotes available from the uteri. These zygotes undergo synchronous development under laboratory conditions [1]. Their prolonged cell cycle (approximately 15 to 20 hours) makes it possible to isolate embryos at precise stages of development [54], including before, during, and after PDE. Although the parasitic lifestyle of *Ascaris* makes maintenance and genetic manipulation difficult, decades of molecular work in *Ascaris* have provided one of the most comprehensive understandings of PDE in a metazoan [6,45,49,50].

Recently, studies in the free-living nematode *Oscheius tipulae* have shown that it undergoes PDE [55,56]. In addition, cytological studies in the *Mesorhabditis* family of nematodes indicate that they also eliminate DNA during embryogenesis [57]. With more complete genomes, rigorous cytological studies, and comparative analysis on the differences in genome coverage between germline and somatic tissues, we predict more organisms with PDE will be identified in the near future [58]. The recent identification of PDE in free-living nematode species prompts questions about how frequently PDE occurs among metazoans (especially nematodes) and the selective advantage(s) it may provide. Free-living nematodes with PDE also present new opportunities to study PDE in genetically tractable systems [56]. These studies will complement work in *Ascaris* and provide comparative and evolutionary perspectives on PDE in metazoans.

### *Ascaris* PDE breaks chromosomes and eliminates DNA

In most multicellular organisms, PDE events occur during early development during the germ-to-soma transition [45,49]. However, the exact timing of PDE varies among species. Cytological data suggest that *P*. *univalens* PDE transpires during the 2 to 32 cell stages, while *Ascaris* PDE occurs during the 4 to 16 cell stages. In both cases, PDE takes place in consecutive early embryo divisions in 5 independent pre-somatic cells (Fig 1B and 1C) [37]. In contrast, PDE in several other systems occurs in many cells at a single embryo division, such as the free-living nematode *O*. *tipulae* (fourth division) [56], the copepod *Mesocyclops edax* (fifth division), and the sea lamprey *Petromyzon marinus* (sixth division) [49]. It is unclear why organisms undergo PDE at different stages of embryogenesis and whether the timing of PDE is strictly associated with the transition from germline to somatic cells.

Cytological studies have revealed elements of chromosome behavior during *Ascaris* elimination mitoses. At metaphase, retained and eliminated DNA are both aligned at the metaphase plate. In early anaphase, the retained DNA segregates to the daughter cells while the eliminated DNA remains at the metaphase plate (Fig 1C). In late anaphase, eliminated DNA breaks down into smaller pieces (Fig 1D). The eliminated DNA stains with an antibody against H3S10P, a histone modification mark associated with condensed chromosomes during mitosis (Fig 1E–1G). Immunogold labeling of H3S10P for electron microscopy (EM) showed that the eliminated DNA is sequestered into double membrane-bound structures during telophase that resemble mammalian micronuclei [59]. The DNA likely undergoes autophagy and degradation in the subsequent cell divisions (Fig 1F and 1G) [41].

What sequences are eliminated during PDE? Early molecular cloning and cytological work showed that a large amount of DNA eliminated in *P*. *univalens* is associated with the HET arms (Fig 1A) that consist of 5- and 10-mer repeats [60]. Likewise, eliminated DNA in *Ascaris* is mainly composed of 121-bp tandem satellite repeats [41,61]. These early observations lead to the assumption that PDE mainly eliminates repeats. However, seminal work indicated that at least 3 single-copy genes are eliminated in *Ascaris* [62–64]. Over the last decade, the *Ascaris* genome was sequenced and improved with multiple iterations using state-of-the-art technologies [38,40,41,65,66]. The latest version was built with long reads (PacBio) and 3D genome conformation data (Hi-C), resulting in 24 fully assembled germline chromosomes [41]. A comparison between the germline and the somatic genome enabled comprehensive identification of eliminated sequences, DNA breaks, and formation of new telomeres during *Ascaris* PDE [41].

**Fig 2:**
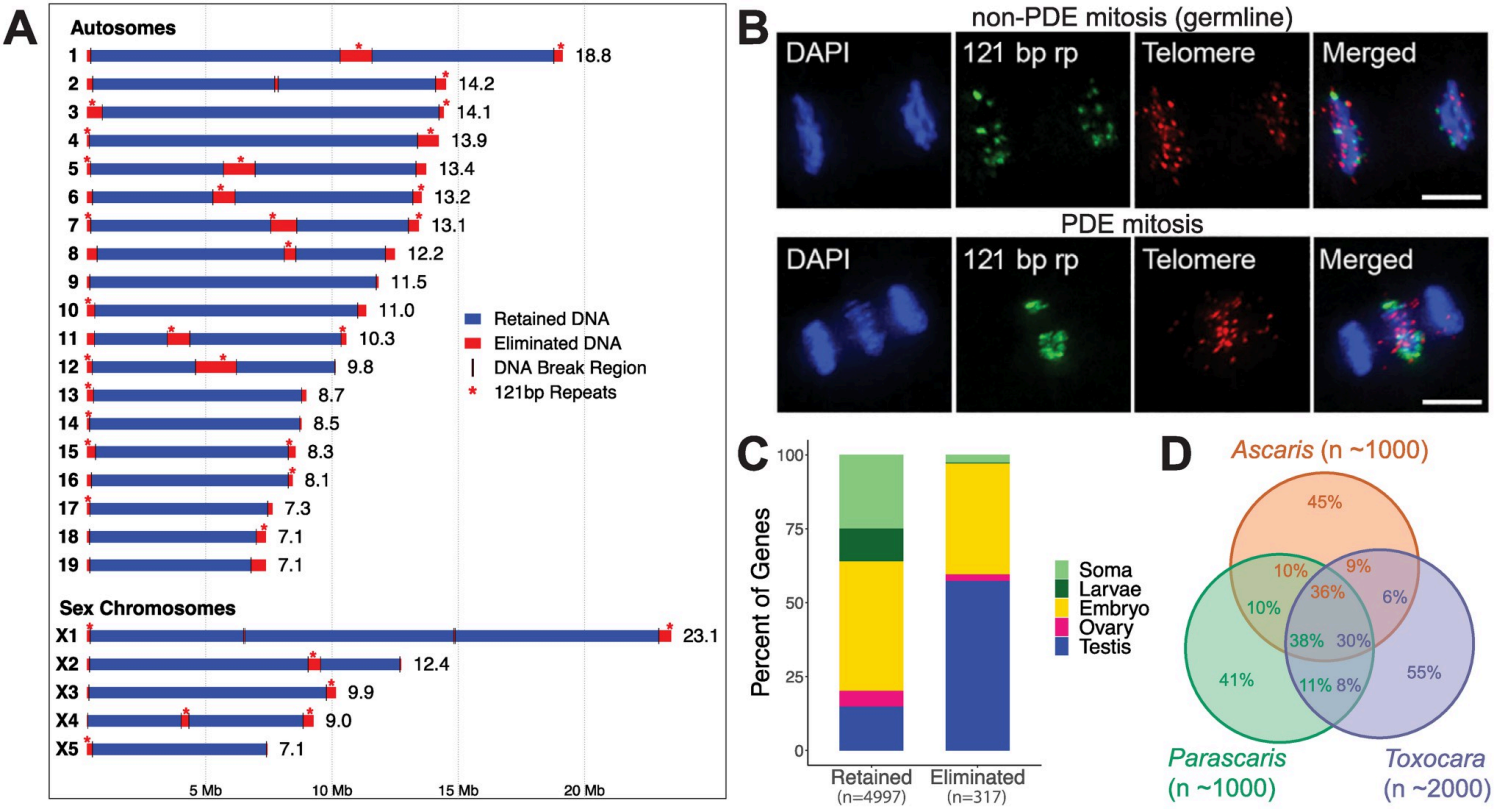
Genetic material lost during PDE. (**A**) A linear presentation of *Ascaris* germline chromosomes. All chromosome ends and 12 internal regions are eliminated (shown in red). Many larger chromosomes have internally eliminated sequences which split them into smaller chromosomes in the soma. Eliminated regions containing 121-bp repeats are marked with a red *. Figure adapted from [6]. (**B**) FISH of 121-bp repeats and telomeric sequences. Top: anaphase in a germline embryo undergoing non-PDE mitosis, with telomeres and 121-bp repeats passing to daughter nuclei. Bottom: anaphase of a PDE mitosis showing 121-bp repeats and telomeres are left at the metaphase plate. (**C**) Eliminated genes are highly enriched with testis and embryo-expressed genes. Shown are changes of the proportion (in percentage, y-axis) of tissue-specific genes in retained and eliminated DNA (x-axis). (**D**) Conservation of eliminated genes among *Ascaris*, *Parascaris*, and *Toxocara*. About 36% of genes eliminated in *Ascaris* have their orthologous genes eliminated in *Parascaris* and *Toxocara*. These genes tend to have enriched expression in the testis. Figure adapted from [6].

Several significant findings emerged from these *Ascaris* genome analyses. First, all germline chromosome ends have a DSB in the subtelomeric region, resulting in the removal and remodeling of each chromosome end (Fig 2A and 2B) [41]. In addition, internal DSBs lead to an increased number of chromosomes from 24 in the germline to 36 in the somatic cells (Fig 2A). No genome rearrangements occur during *Ascaris* PDE [41]. Second, approximately 1,000 (5% of the total) genes are present in the eliminated DNA. These genes are expressed in the germline, primarily during spermatogenesis (Fig 2C) [40,41]. Their elimination from the somatic cells suggests that PDE in *Ascaris* is an extreme gene silencing mechanism [38,40]. Third, telomeres are added to the broken DNA ends. The telomere addition sites occur within a 3 to 6 kb region called chromosomal breakage region (CBR). The CBRs appear to have no common motif or sequence features (see below), suggesting a sequence-independent mechanism of break site recognition [40]. Last, the CBRs and eliminated sequences are consistent among the 5 independent pre-somatic cells, between male and female worms, and between the human (*A*. *lumbricoides*) and pig (*A*. *suum*) parasites [40].

Most ascarid species undergo PDE [37]. How conserved is PDE among ascarids? A comparative genomic study identified approximately 1,000 eliminated genes in the horse parasite *P*. *univalens* and approximately 2,000 in the dog parasite *T*. *canis* [40]. About one-third of the eliminated genes are conserved among these 2 species and *Ascaris* (Fig 2D). The conserved, eliminated genes in all 3 species are primarily expressed during spermatogenesis, suggesting a common mechanism for silencing testis genes in the somatic cells. Interestingly, the amount and composition of eliminated repetitive sequences varies drastically among the 3 species. *Ascaris* eliminates 121-bp repeats (10% of the genome) (Fig 2A and 2B), while *P*. *univalens* removes 5-bp and 10-bp repeats (89% of the genome), and *T*. *canis* throws away 49-bp satellite sequences (9% of the genome) [40]. The differences in tandem repeat composition may reflect their different origins and/or species-specific adaptions during nematode evolution.

In summary, *Ascaris* undergoes PDE in all 5 pre-somatic cells during the 4 to 16 cell stages. During PDE, 72 DSBs are introduced at specific sites (Fig 2A). The DSBs remove all chromosome ends and split germline chromosomes into smaller somatic chromosomes, thus increasing the number of chromosomes in the soma. During this process, approximately 18% of the germline genome is lost. The eliminated DNA is composed of satellite repeats and contains approximately 1,000 genes. Following DSB formation, all broken ends, including the ends of eliminated sequences, are healed with de novo telomere addition.

### Molecular mechanisms of *Ascaris* PDE

What mechanisms are involved in PDE and how is the process regulated? Boveri noted that after centrifugation of *P*. *equorum* embryos, a “ball” of cytoplasmic factors would form at the animal pole. The 2 blastomeres proximal to this ball would undergo PDE, while the 2 blastomeres distal to the ball would not. This led to the hypothesis of a cytoplasmic factor that induces PDE [44]. Subsequent studies by Moritz showed that UV irradiation of *Parascaris* embryos resulted in all cells undergoing PDE [44,67]. This suggests that a UV-sensitive factor is responsible for inhibiting PDE in germline cells. Experiments by Oliver and Shen, using di-nucleated *Ascaris* embryos obtained by treatment with cytochalasin D (to prevent cytokinesis), further demonstrated that 1 blastomere always undergoes PDE while the other never does [68]. Together, these early experiments suggest a complex regulatory system where PDE-promoting factors associate with pre-somatic cells and PDE-inhibiting factors associate with germ cells [68]. These regulatory factors have yet to be identified.

**Fig 3:**
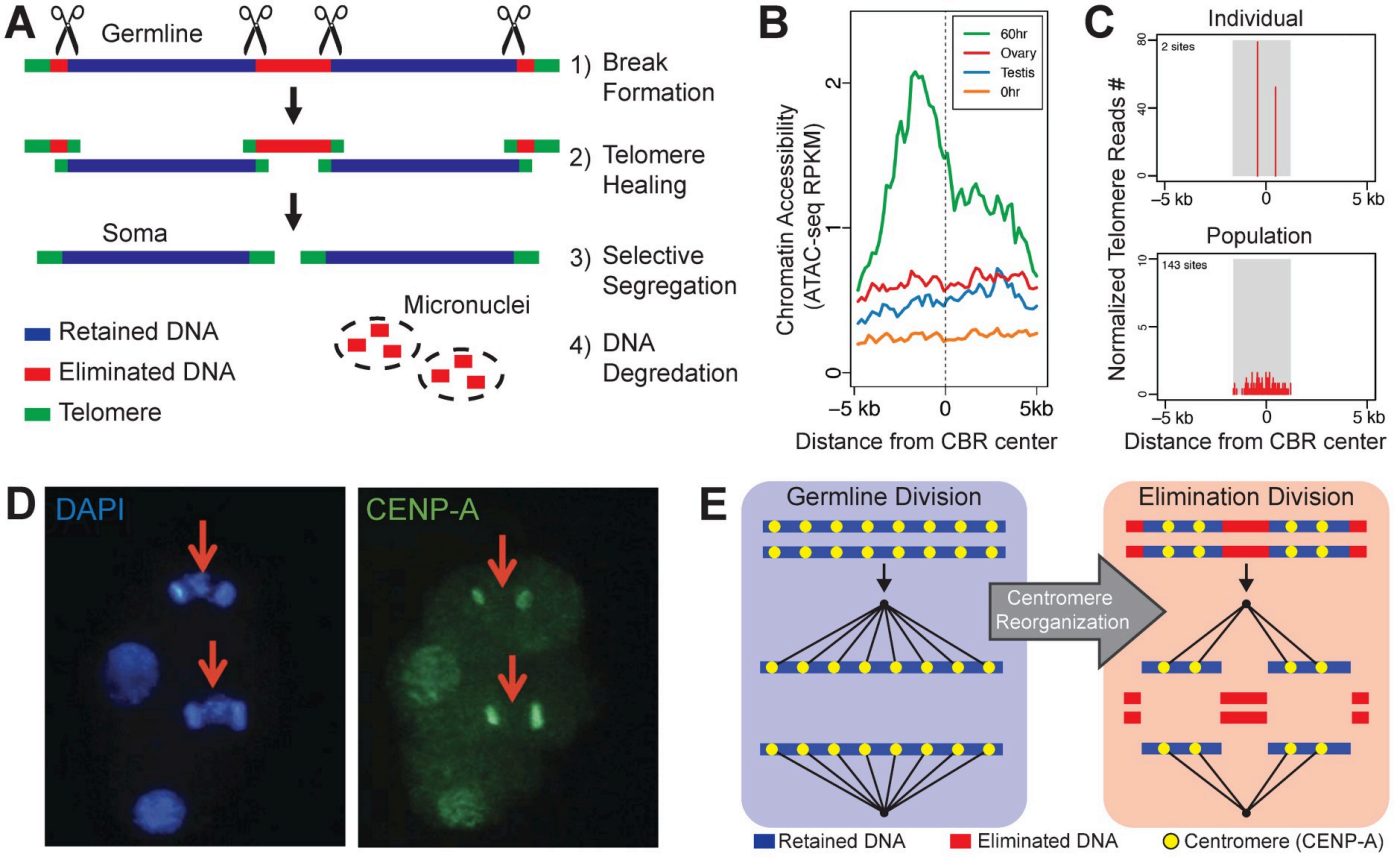
PDE mechanisms. (**A**) A model of PDE process in *Ascaris*. See text for detailed description. (**B**) ATAC-seq of *Ascaris* shows CBRs have open chromatin during PDE (60 hours, 4-cell). (**C**) *Ascaris* telomere addition forms in 3–6-kb regions of the genome. Top: telomere addition from a single worm’s intestine (derived from 1 PDE event) shows 2 telomere addition sites. Bottom: telomere addition from a population of worms shows telomeres are randomly distributed across the CBR. (**B**) and (**C**) adapted from [40]. (**D**) CENP-A staining of a 4-cell embryo with 2 cells undergoing PDE. Eliminated DNA (red arrows) is not stained with CENP-A, suggesting the loss of centromeres in the eliminated DNA. Adapted from [39]. (**E**) A model of centromere reorganization for PDE. Left: germline division with centromeres distributed across the entire chromosome. Right: PDE mitosis with centromeres absent on eliminated regions of the genome. Adapted from [39].

At the molecular level, the process of PDE in *Ascaris* can be summarized in 4 major steps: (1) recognition of the sites for DNA breakage and generation of chromosome breaks; (2) healing broken DNA ends by telomere addition; (3) selective segregation of retained DNA; and (4) degradation of eliminated DNA (Fig 3A). It’s not clear if these steps are coupled or if they are independent, sequential events. Overall, little is known about the molecular details of *Ascaris* PDE. Below, we summarize what is known and discuss possible molecular mechanisms involved in these steps.

*How are break sites recognized and generated*? Analysis of break regions for the presence of specific DNA sequences, structural motifs, palindromes, Z-DNA, and hairpins have revealed no apparent features that define or target CBRs for PDE break formation [41]. Additionally, no small RNAs or long non-coding RNAs appear to directly mark the break sites or contribute to PDE [66,69]. A recent analysis suggested that G-quadruplex DNA is enriched in extended (10 to 20 kb) regions covering 40 *Ascaris* CBRs [70]. However, G-quadruplexes are also enriched in other regions of the *Ascaris* genome, and many CBRs have no G-quadruplexes [70], suggesting G-quadruplex DNA is probably not a component of the CBRs.

One consistent feature of *Ascaris* CBRs is the increased chromatin accessibility before and during PDE, as measured by ATAC-seq (Fig 3B) [40,41]. This accessibility may play a significant role in *Ascaris* PDE. In many systems, replication stress, transcription, and R-loop formation facilitate chromatin opening [71–73]. In addition, these factors are a source of genome instability and could lead to DSB formation [74]. Therefore, it is tempting to speculate that replication and transcription may be responsible for chromatin opening and DSB formation at *Ascaris* CBRs. The open chromatin may allow an endonuclease to access the CBR to generate the DSBs. In the free-living nematode *O*. *tipulae*, a conserved motif is necessary for the DNA break and telomere addition required for PDE [56]. In contrast, *Ascaris* CBRs have no common motif, thus a sequence-independent mechanism, such as 3D genome organization [75–77], may be needed to confine break formation to the CBRs.

*How are the ends processed after a DNA break and how are telomeres added*? Interestingly, genome sequencing indicates that 2 independent telomere addition sites are often observed in a single PDE event; the 2 sites are presumably from the 2 homologous chromosomes (Fig 3C) [40,78,79]. In a population of *Ascaris* worms, telomere addition sites are randomly distributed within a constrained 3- to 6-kb region, which defines the CBRs (Fig 3C). This suggests the telomere addition sites are sequence-independent and random within the restricted genomic regions. Remarkably, telomere addition occurs not only to retained DNA but also to eliminated DNA [40,78,79]. This indicates that telomere addition in *Ascaris* could be non-selective; it may heal all available PDE DNA breaks. This universal healing likely protects the broken DNA ends from undergoing fusions and genome rearrangements [36]. Analysis of these telomere addition sites has revealed that a single nucleotide of homology is sufficient for telomere addition in vivo [40]. Since the *Ascaris* telomeric repeat (TTAGGC) contains all 4 bases, the requirement of a single nucleotide means telomere addition can occur at any site within the CBRs. Telomerase activity from *Ascaris* cell-free extracts increases 10-fold from the one-cell to four-cell stage and remains elevated past the final PDE event [40,80]. This is consistent with RNA-seq data [81] and suggests telomerase is likely responsible for the synthesis of de novo telomeres during *Ascaris* PDE.

The telomere addition site is not necessarily the site where the initial DSB occurs. DSBs could occur at multiple sites within eliminated regions, adjacent to the CBRs followed by end resection, and/or at the site of telomere addition. Estrem and colleagues used END-seq, a technique that captures DSBs and end resections [82,83], to identify the position and timing of DNA breaks on synchronized embryos from before and during PDE stages [Estrem, Davis, and Wang, personal communications]. END-seq data identified that extensive resection occurs to generate 3′-overhangs necessary for telomere addition. This end resection is also conserved in *O*. *tipulae* PDE [56]. In addition, the timing of initial breaks detected by END-seq suggests *Ascaris* chromosomes are broken prior to the onset of mitosis, consistent with EM observations [41]. This indicates that cellular mechanisms are required to congress eliminated chromosome fragments to the metaphase plate during DNA elimination mitosis (see Fig 1).

*How is retained DNA recognized and selectively segregated after DSBs*? Immunohistochemical (IHC) analysis of histone marks (H3K4me1/3, H3K9me2/3, H3K36me2/3, H4K16ac, and H3K27me3) revealed no differences between retained and eliminated DNA in anaphase during PDE [41]. However, H3S10P is enriched on eliminated DNA (Fig 1E) and H2AK119ub is associated with retained DNA [41]. In addition, 2 worm-specific Argonaute proteins (WAGOs) associated with endogenous small RNA (siRNA) pathways are differentially enriched in retained (WAGO-2) and eliminated (WAGO-3) DNA during PDE mitosis [Zagoskin, Wang, and Davis, personal communications]. It is unclear if these histone modifications or the WAGO argonautes are actively marking sequences for their retention/elimination or if they are an indirect consequence of PDE. For example, H3S10P on the eliminated DNA is likely due to a failure to dephosphorylate H3S10P as the eliminated DNA fragments are compartmentalized into micronuclei and may not progress through the normal metaphase to anaphase transition [41] (Fig 1F and 1G).

After the fragmentation of the chromosomes, how does the cell decide which chromosome pieces to keep and discard? Like the model nematode *Caenorhabditis elegans*, the chromosomes in *Ascaris* and *P*. *univalens* are holocentric, with multiple centromeres/kinetochores distributed along the length of the chromosomes, a feature that in principle makes DNA breakage more tolerable [39,84,85]. Staining data demonstrated that during PDE, the histone H3 variant CENP-A, the epigenetic marker of centromeres, is absent in the DNA to be eliminated (Fig 3D). This lack of centromeres in the eliminated DNA provides a mechanism for its subsequent loss after the DNA breaks, as no microtubules can attach to the DNA to facilitate its proper segregation during mitosis. ChIP-seq showed that CENP-A is evenly distributed across the genome in *Ascaris* male and female germlines [39]. These data suggest a dynamic reorganization of centromere deposition that enables *Ascaris* to selectively segregate the retained DNA into daughter nuclei while excluding the eliminated DNA (Fig 3D and 3E) [39]. The regulation of centromere reorganization has not been elucidated in *Ascaris*. However, transcription, histone modifications, and small RNAs have been linked to dynamic centromere deposition in fission yeast and nematodes [86–88].

*How is eliminated DNA degraded*? The enrichment of H3S10P on eliminated DNA provided a mechanism to track this chromatin following mitosis. H3S10P co-localizes with LGG1, an autophagosome marker [41]. Additionally, immuno-electron microscopy of H3S10P revealed many double membrane-bound structures are positive for H3S10P during the telophase following PDE (Fig 1F and 1G) [41]. These results suggest that, following mitosis, eliminated DNA is autophagocytosed into micronuclei-like structures. Large heterochromatic regions may take a few cell cycles to completely turn over, as remnants of the eliminated DNA from previous cell cycles are often seen in subsequent developmental stages (Fig 1D). The engulfment of the eliminated DNA into micronuclei may block the DNA from unnecessary repair and/or recombination events. Recent work has shown that micronuclei are prone to rupture, resulting in DNA fragmentation [89]. The mechanism of micronuclei fragmentation is not well understood, but aberrant replication or exposure to nucleases in the cytoplasm may drive this process [89–91]. Nematodes with PDE may provide a new model to study DNA fragmentation in micronuclei. For example, it will be interesting to determine if the DNA to be eliminated in *Ascaris* is turned over through an orderly or random process.

### Functions of *Ascaris* PDE

The presence of PDE in phylogenetically diverse organisms suggests PDE may provide some selective advantage [43,45,49]. One consequence of PDE is the permanent removal of specific repetitive elements in all somatic cells, a common feature among all known species that undergo PDE [40,55,56]. In *Ascaris*, 54% of the eliminated DNA consists of 121 bp tandem repeats. In addition, almost all satellite repeats (>99%) are eliminated from *Ascaris*, *P*. *univalens*, and *T*. *canis* [40]. These repeats likely have germline functions that are not needed (or are harmful) in the somatic cells. These functions could be related to meiotic chromosome pairing and recombination, spacing and scaffolding of 3D genome organization in the germline, and evolution of novel genes and/or chromosomes [41,45,92–95]. Another benefit of removing repeats, particularly large amounts of satellites such as the 89% (2.2 Gb) eliminated from *P*. *univalens*, could be to downsize the genome, alleviating the burden of DNA replication and genome maintenance in somatic cells.

Another advantage of PDE could be the removal of germline-specific genes. Typically, germline genes in nematodes are silenced by histone modifications and small RNAs [96–98]. In *Ascaris*, most of the approximately 1,000 eliminated genes are expressed in the testis, ovary, or during early embryogenesis (Fig 2C) [38,40]. Thus, PDE provides a permanent means to silence these genes in the somatic cells. The removal of testis-expressed genes is conserved in the ascarids *P*. *univalens* and *T*. *canis* [6,40], further supporting the role of silencing germline-expressed genes for PDE in ascarids. This gene silencing appears to be a common theme for PDE as germline-specific genes are identified in phylogenetically diverse species that undergo PDE [40,43,45,49]. In *Ascaris*, many of the eliminated genes have paralogs in the genome, including a germline-specific ribosomal protein [62,99] and translation initiation factors that are involved in mRNA translation [38,99]. Duplication of genes provides an alternative means of gene regulation. This is consistent with the model that an ancient genome duplication in the *Ascaris* lineage was balanced by PDE to compensate for gene dosage or to retain specific genes and thus their function after gene duplication [38].

What is the benefit of chromosome end removal and remodeling? Repetitive DNA and novel genes are often enriched at the ends of the nematode chromosomes, while more conserved and housekeeping genes are concentrated in the middle of the chromosomes [100]. All 13 *Ascaris* germline chromosomes with no internal PDE breaks have this sequence organization [41]. The other 11 germline chromosomes are believed to be potential fusions of multiple ancestral chromosomes (see below section on the evolution of PDE). Removal of repeats and novel genes from chromosome ends may protect the soma from potentially harmful sequences while allowing their existence in the germline. This may provide an evolutionary advantage for the germline to sample novel genes or harbor repeats. An alternative hypothesis is chromosome ends act as pairing centers that are needed in the germline but deleterious to the somatic cells. Consistent with this notion, proteins involved in *C*. *elegans* meiotic pairing [92–95] appear absent from *Ascaris*’s genome [Wang, personal communications]. A recent study in the free-living nematode *O*. *tipulae* demonstrated a fail-safe mechanism is in place to ensure the removal of chromosome ends during PDE [56], emphasizing the importance of end removal for the biology of these species. Future studies are warranted to determine the functions of the eliminated DNA in the germline and the benefit of its removal in the somatic cells.

PDE clearly poses advantages in *Ascaris* and other nematodes. It is a mechanism to silence germline sequences and repetitive elements in the somatic genome. End removal and remodeling appear conserved among nematodes. In addition, PDE can alter the somatic chromosome karyotype, possibly leading to changes in the 3D genome organization and gene expression in the soma. These functions may provide an additional benefit to the survival and fitness of the organisms.

### Evolution of PDE in nematodes

**Fig 4:**
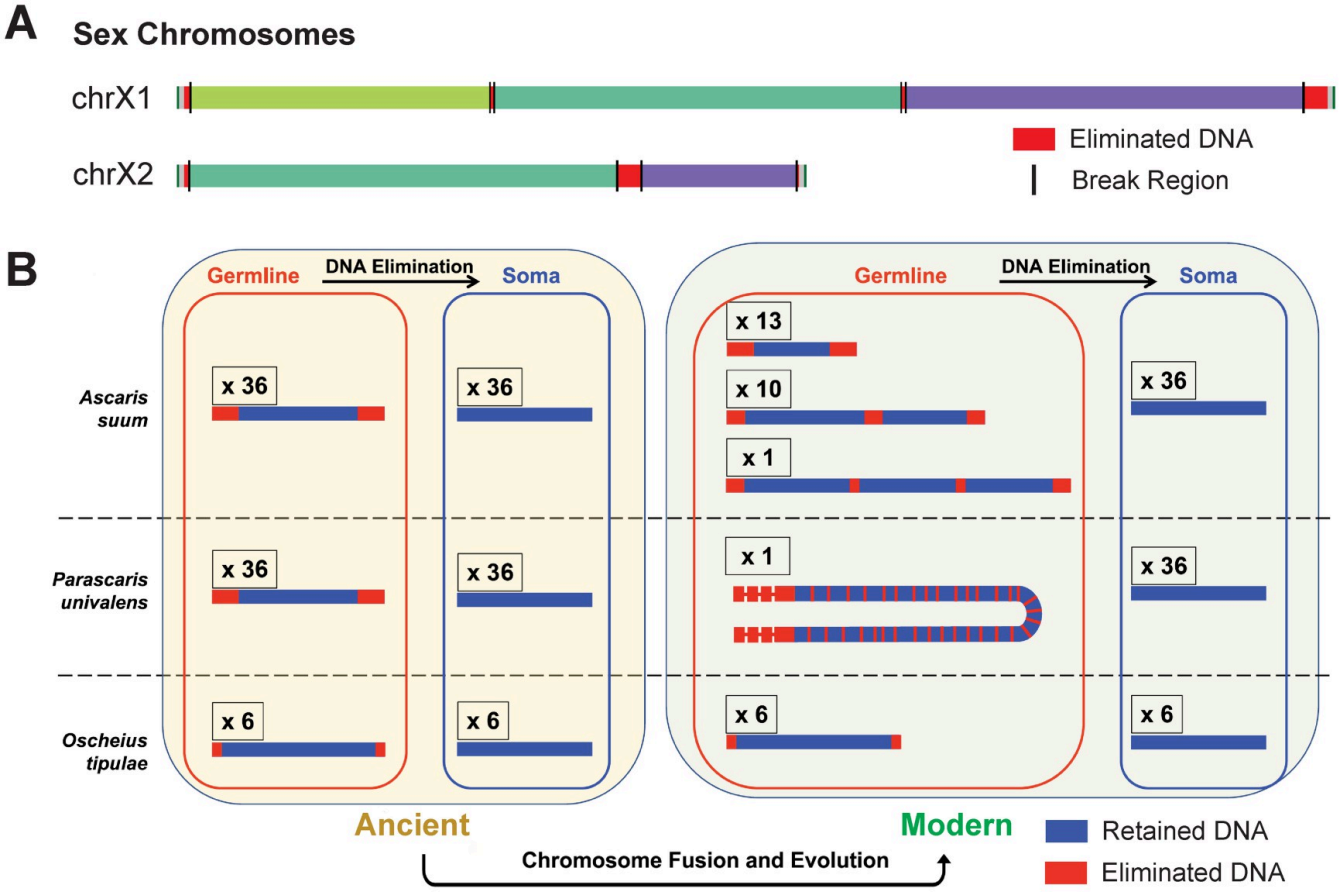
An evolution model of PDE in nematode chromosomes. (**A**) Fusions in *Ascaris* sex chromosomes. Two representative sex chromosomes with distinct ancestral regions (Nigon elements; each Nigon group is shown in a different color). Eliminated DNA is at the junction of different Nigon elements. (**B**) Fusion and evolution of chromosomes in nematodes with PDE. Left: hypothetical ancient chromosome karyotypes before and after chromosome end remodeling and DNA elimination in *Ascaris*, *P*. *univalens*, and *O*. *tipulae*. Right: modern karyotypes before and after DNA elimination showing internal breaks and end-remodeling. For simplicity, a representative chromosome is shown for all chromosomes within a group that have the same pattern of elimination (terminal and/or internal). The number of chromosomes for each group is indicated above the representative chromosome. (**A**) and (**B**) are adapted from [45].

Comparative analysis of the *Ascaris* genome has revealed that 4 out of 5 sex chromosomes have undergone recent fusion events. These sex chromosomes are chimeric in their composition of Nigon elements (Fig 4A) [41,55]. Interestingly, the internally eliminated DNA represents the junction of presumptive fusion events on these sex chromosomes [41,55]. PDE splits these fused germline chromosomes into separate chromosomes in the somatic cells. Analysis of the fully assembled *P*. *univalens* germline and somatic genomes reveals that it has the same number (36) of somatic chromosomes as *Ascaris* [Simmons and Wang, personal communications]. The internally eliminated regions in both *Ascaris* and *Parascaris* have sequence features that are common to the ends of nematode chromosomes [85], including their enrichment of repeats, genes encoding hypothetical proteins, and genes specifically expressed in the testis. This suggests that these internal eliminated regions were likely originated from the ends of ancestral chromosomes. Thus, the current single germline chromosome of *Parascaris* and the 24 *Ascaris* germline chromosomes can be viewed as fusion products of the 36 ancient germline chromosomes. These ancestral germline chromosomes also likely underwent PDE with end remodeling (Fig 4B).

PDE in *Ascaris* and *Parascaris* provides a way to resolve these fused germline chromosomes to form the 36 somatic chromosomes. The reduced number of chromosomes in the germline should decrease the probability of segregation errors during meiosis. In comparison, the smaller somatic chromosomes may be more adapted to the conventional gene expression regulation in nematode, as the organization of the genes and repeats in these chromosomes resembles a typical nematode chromosome [85]. Thus, both fused germline chromosomes and split somatic chromosomes may pose advantages. Recent data from some free-living nematodes indicates internal breaks also occur [85]. This finding does not preclude PDE as a mechanism to resolve chromosome fusions in ascarids. Instead, it suggests that de novo introduction of DNA breaks in the middle of the chromosomes is possible, likely enabled by the holocentric nature of nematode chromosomes [85].

In nematodes, PDE has been discovered in 3 distinct clades, including many *Oscheius* species [55,56], *Caenorhabditis monodelphis*, and *Mesorhabditis* [57] from Clade V, ascarids (*Ascaris*, *Parascaris Toxocara*, and *Baylisascaris*) from Clade III [37], and *Strongyloides* [35,101,102] from Clade IV. A common theme among *Ascaris*, *P*. *univalens*, and *O*. *tipulae* PDE is the removal of all chromosome ends, followed by new telomere addition. This leads to the question whether PDE is an ancient trait, or if it has convergently evolved in multiple nematode clades. It is known that the model organism *C*. *elegans* (Clade V) does not undergo PDE [103,104]. But how common is PDE among nematodes? Other nematodes may also remodel their chromosome ends with PDE, but the removal of only small amounts of DNA may have eluded detection due to technical limitations such as the sensitivity of cytological approaches and/or the requirement of telomere-to-telomere genome assemblies [55]. PDE may be more common or favored in nematodes because the holocentric nature of their chromosomes that allows fusions and fissions to be tolerated [105,106]. However, holocentromeres are not required for PDE as it is seen in several phyla with monocentric chromosomes [36,45,49]. Nevertheless, end-remodeling appears to be a form of PDE that is often missed, suggesting PDE may be much more frequent than previously thought. Future identification of additional species with PDE will further elucidate the evolutionary relationship of PDE in various organisms.

### Perspective

PDE violates the general view of genome constancy and integrity. However, PDE is a natural process found in many branches of animal phyla, suggesting its common presence and biological significance. The number of species described with PDE has increased with the use of genome sequencing and modern cytology. However, mechanistic insights into PDE are mostly limited to single-cell ciliates. For metazoans, the nematode group has been a leading model to study PDE since its discovery over 130 years ago. In particular, the large size of *Ascaris*, the ability to dissect specific germline and somatic tissues, and its slow, synchronous embryo development have enabled robust biochemistry, molecular biology, and genomics to be carried out. Other parasitic ascarids, including *Parascaris* and *Toxocara*, have also been used as comparative models to study PDE. Many features of PDE, including DNA breaks, telomere addition, and chromosome end removal and remodeling, are conserved in these nematodes [6,40]. The newly established PDE model in the free-living nematode *O*. *tipulae* now adds genetic and functional approaches to study PDE [56]. Overall, these studies will unveil the functions and mechanisms of PDE in nematodes, including the human parasite *Ascaris*.

## References

[1] Wang J, Davis RE. Ascaris. Curr Biol. 2020;30(10):R423–R5. Epub 2020/05/20. doi: 10.1016/j.cub.2020.02.064 . 32428467

[2] Easton A, Gao S, Lawton SP, Bennuru S, Khan A, Dahlstrom E, et al. Molecular evidence of hybridization between pig and human Ascaris indicates an interbred species complex infecting humans. Elife. 2020;9. Epub 2020/11/07. doi: 10.7554/eLife.61562 33155980 PMC7647404

[3] Miller LA, Colby K, Manning SE, Hoenig D, McEvoy E, Montgomery S, et al. Ascariasis in humans and pigs on small-scale farms, Maine, USA, 2010–2013. Emerg Infect Dis. 2015;21(2):332–4. doi: 10.3201/eid2102.140048 25626125 PMC4313629

[4] Betson M, Nejsum P, Bendall RP, Deb RM, Stothard JR. Molecular epidemiology of ascariasis: a global perspective on the transmission dynamics of Ascaris in people and pigs. J Infect Dis. 2014;210(6):932–41. Epub 20140331. doi: 10.1093/infdis/jiu193 24688073 PMC4136802

[5] Sadaow L, Sanpool O, Phosuk I, Rodpai R, Thanchomnang T, Wijit A, et al. Molecular identification of Ascaris lumbricoides and Ascaris suum recovered from humans and pigs in Thailand, Lao PDR, and Myanmar. Parasitol Res. 2018;117(8):2427–36. Epub 20180602. doi: 10.1007/s00436-018-5931-6 . 29860571

[6] Wang J. Genomics of the Parasitic Nematode Ascaris and Its Relatives. Genes (Basel). 2021;12(4). Epub 2021/04/04. doi: 10.3390/genes12040493 33800545 PMC8065839

[7] Holland C, Sepidarkish M, Deslyper G, Abdollahi A, Valizadeh S, Mollalo A, et al. Global prevalence of Ascaris infection in humans (2010–2021): a systematic review and meta-analysis. Infect Dis Poverty. 2022;11(1):113. Epub 20221118. doi: 10.1186/s40249-022-01038-z 36401308 PMC9673379

[8] Bethony J, Brooker S, Albonico M, Geiger SM, Loukas A, Diemert D, et al. Soil-transmitted helminth infections: ascariasis, trichuriasis, and hookworm. Lancet. 2006;367(9521):1521–32. doi: 10.1016/S0140-6736(06)68653-4 . 16679166

[9] Hotez PJ, Brindley PJ, Bethony JM, King CH, Pearce EJ, Jacobson J. Helminth infections: the great neglected tropical diseases. J Clin Invest. 2008;118(4):1311–21. doi: 10.1172/JCI34261 18382743 PMC2276811

[10] Jourdan PM, Lamberton PHL, Fenwick A, Addiss DG. Soil-transmitted helminth infections. Lancet. 2018;391(10117):252–65. Epub 20170904. doi: 10.1016/S0140-6736(17)31930-X . 28882382

[11] Brooker S. Estimating the global distribution and disease burden of intestinal nematode infections: adding up the numbers—a review. Int J Parasitol. 2010;40(10):1137–44. Epub 20100427. doi: 10.1016/j.ijpara.2010.04.004 20430032 PMC3034165

[12] Matthews JB. Anthelmintic resistance in equine nematodes. Int J Parasitol Drugs Drug Resist. 2014;4(3):310–5. Epub 2014/12/18. doi: 10.1016/j.ijpddr.2014.10.003 25516842 PMC4266799

[13] Adugna S, Kebede Y, Moges F, Tiruneh M. Efficacy of mebendazole and albendazole for Ascaris lumbricoides and hookworm infections in an area with long time exposure for antihelminthes, Northwest Ethiopia. Ethiop Med J. 2007;45(3):301–6. Epub 2008/03/12. . 18330331

[14] Nareaho A, Vainio K, Oksanen A. Impaired efficacy of ivermectin against Parascaris equorum, and both ivermectin and pyrantel against strongyle infections in trotter foals in Finland. Vet Parasitol. 2011;182(2–4):372–7. Epub 2011/06/22. doi: 10.1016/j.vetpar.2011.05.045 . 21689886

[15] Mohammed SH, Jabbr AS, Ibrahim NK. Impact of parasitic infection with Ascaris lumbricoides on pulmonary function tests in asthmatic and non-asthmatic children. Respir Med Case Rep. 2021;34:101552. Epub 20211110. doi: 10.1016/j.rmcr.2021.101552 34820258 PMC8600146

[16] Pereira de Araujo M, Sato MO, Sato M, Bandara Wm KM, Coelho LFL, Souza RLM, et al. Unbalanced relationships: insights into the interaction between gut microbiota, geohelminths, and schistosomiasis. PeerJ. 2022;10:e13401. Epub 20220505. doi: 10.7717/peerj.13401 35539016 PMC9080432

[17] Longo FJ. Fertilization: a comparative ultrastructural review. Biol Reprod. 1973;9(2):149–215. doi: 10.1093/biolreprod/9.2.149 . 4583963

[18] Singaravelu G, Singson A. New insights into the mechanism of fertilization in nematodes. Int Rev Cell Mol Biol. 2011;289:211–38. doi: 10.1016/B978-0-12-386039-2.00006-7 21749902 PMC3273857

[19] Maderspacher F. Theodor Boveri and the natural experiment. Curr Biol. 2008;18(7):R279–86. Epub 2008/04/10. doi: 10.1016/j.cub.2008.02.061 . 18397731

[20] Satzinger H. Theodor and Marcella Boveri: chromosomes and cytoplasm in heredity and development. Nat Reviews Genet. 2008;9(3):231–8. Epub 2008/02/13. doi: 10.1038/nrg2311 . 18268510

[21] Hannon GJ, Maroney PA, Denker JA, Nilsen TW. Trans splicing of nematode pre-messenger RNA in vitro. Cell. 1990;61(7):1247–55. Epub 1990/06/29. doi: 10.1016/0092-8674(90)90689-c . 2163760

[22] Davis RE. Spliced leader RNA trans-splicing in metazoa. Parasitol Today. 1996;12(1):33–40. doi: 10.1016/0169-4758(96)80643-0 . 15275306

[23] Lall S, Friedman CC, Jankowska-Anyszka M, Stepinski J, Darzynkiewicz E, Davis RE. Contribution of trans-splicing, 5’ -leader length, cap-poly(A) synergism, and initiation factors to nematode translation in an Ascaris suum embryo cell-free system. J Biol Chem. 2004;279(44):45573–85. Epub 2004/08/24. doi: 10.1074/jbc.M407475200 . 15322127

[24] Cohen LS, Mikhli C, Friedman C, Jankowska-Anyszka M, Stepinski J, Darzynkiewicz E, et al. Nematode m7GpppG and m3(2,2,7)GpppG decapping: activities in Ascaris embryos and characterization of C. elegans scavenger DcpS. RNA. 2004;10(10):1609–24. Epub 2004/09/24. doi: 10.1261/rna.7690504 15383679 PMC1370647

[25] Davis RE, Stretton AO. The motornervous system of Ascaris: electrophysiology and anatomy of the neurons and their control by neuromodulators. Parasitology. 1996;113 Suppl:S97–117. Epub 1996/01/01. doi: 10.1017/s0031182000077921 . 9051930

[26] Stretton AO, Maule AG. The Neurobiology of Ascaris and Other Parasitic Nematodes. Ascaris: The Neglected Parasite. 2013:127–52.

[27] Rathbone L. Oxidative metabolism in Ascaris lumbricoides from the pig. Biochem J. 1955;61(4):574–9. Epub 1955/12/01. doi: 10.1042/bj0610574 13276339 PMC1215836

[28] Beis I, Barrett J. Energy metabolism in developing Ascaris lumbricoides eggs. II, The steady state content of intermediary metabolites. Dev Biol. 1975;42(1):188–95. Epub 1975/01/01. doi: 10.1016/0012-1606(75)90323-1 . 1167531

[29] Barrett J, Beis I. Energy metabolism in developing Ascaris lumbricoides eggs. I. The glycolytic enzymes. Dev Biol. 1975;42(1):181–7. Epub 1975/01/01. doi: 10.1016/0012-1606(75)90322-x . 1167530

[30] Tan JH, Lautens M, Romanelli-Cedrez L, Wang J, Schertzberg MR, Reinl SR, et al. Alternative splicing of coq-2 controls the levels of rhodoquinone in animals. Elife. 2020;9. Epub 2020/08/04. doi: 10.7554/eLife.56376 32744503 PMC7434440

[31] Theriot JA. Worm sperm and advances in cell locomotion. Cell. 1996;84(1):1–4. Epub 1996/01/12. doi: 10.1016/s0092-8674(00)80068-9 . 8548813

[32] Italiano JE Jr., Roberts TM, Stewart M, Fontana CA. Reconstitution in vitro of the motile apparatus from the amoeboid sperm of Ascaris shows that filament assembly and bundling move membranes. Cell. 1996;84(1):105–14. Epub 1996/01/12. doi: 10.1016/s0092-8674(00)80997-6 . 8548814

[33] Bottino D, Mogilner A, Roberts T, Stewart M, Oster G. How nematode sperm crawl. J Cell Sci. 2002;115(Pt 2):367–84. Epub 2002/02/13. doi: 10.1242/jcs.115.2.367 . 11839788

[34] Roberts TM, Stewart M. Role of major sperm protein (MSP) in the protrusion and retraction of Ascaris sperm. Int Rev Cell Mol Biol. 2012;297:265–93. Epub 2012/05/23. doi: 10.1016/B978-0-12-394308-8.00007-8 . 22608562

[35] Streit A, Wang J, Kang Y, Davis RE. Gene silencing and sex determination by programmed DNA elimination in parasitic nematodes. Curr Opin Microbiol. 2016;32:120–7. Epub 2016/06/18. doi: 10.1016/j.mib.2016.05.012 27315434 PMC4983467

[36] Wang J, Davis RE. Programmed DNA elimination in multicellular organisms. Curr Opin Genet Dev. 2014;27:26–34. Epub 2014/06/03. doi: 10.1016/j.gde.2014.03.012 24886889 PMC4125452

[37] Muller F, Tobler H. Chromatin diminution in the parasitic nematodes ascaris suum and parascaris univalens. Int J Parasitol. 2000;30(4):391–9. Epub 2000/03/25. doi: 10.1016/s0020-7519(99)00199-x . 10731562

[38] Wang J, Mitreva M, Berriman M, Thorne A, Magrini V, Koutsovoulos G, et al. Silencing of germline-expressed genes by DNA elimination in somatic cells. Dev Cell. 2012;23(5):1072–80. Epub 2012/11/06. doi: 10.1016/j.devcel.2012.09.020 23123092 PMC3620533

[39] Kang Y, Wang J, Neff A, Kratzer S, Kimura H, Davis RE. Differential Chromosomal Localization of Centromeric Histone CENP-A Contributes to Nematode Programmed DNA Elimination. Cell Rep. 2016;16(9):2308–16. Epub 2016/08/23. doi: 10.1016/j.celrep.2016.07.079 27545882 PMC5007152

[40] Wang J, Gao S, Mostovoy Y, Kang Y, Zagoskin M, Sun Y, et al. Comparative genome analysis of programmed DNA elimination in nematodes. Genome Res. 2017;27(12):2001–14. Epub 2017/11/10. doi: 10.1101/gr.225730.117 29118011 PMC5741062

[41] Wang J, Veronezi GMB, Kang Y, Zagoskin M, O’Toole ET, Davis RE. Comprehensive Chromosome End Remodeling during Programmed DNA Elimination. Curr Biol. 2020;30(17):3397–413 e4. Epub 2020/07/18. doi: 10.1016/j.cub.2020.06.058 32679104 PMC7484210

[42] Boveri T. Ueber Differenzierung der Zellkerne wahrend der Furchung des Eies von Ascaris megalocephala. Anat Anz. 1887;2:688–93.

[43] Dedukh D, Krasikova A. Delete and survive: strategies of programmed genetic material elimination in eukaryotes. Biol Rev Camb Philos Soc. 2021. Epub 2021/09/21. doi: 10.1111/brv.12796 . 34542224 PMC9292451

[44] Tobler H. The differentiation of germ and somatic cell lines in nematodes. Results Probl Cell Differ. 1986;13:1–69. Epub 1986/01/01. doi: 10.1007/978-3-540-39838-7_1 . 3018878

[45] Zagoskin M, Wang J. Programmed DNA elimination: silencing genes and repetitive sequences in somatic cells. Biochem Soc Trans. 2021. doi: 10.1042/BST20190951 34665225 PMC9200590

[46] Goto Y, Kubota S, Kohno S. Highly repetitive DNA sequences that are restricted to the germ line in the hagfish Eptatretus cirrhatus: a mosaic of eliminated elements. Chromosoma. 1998;107(1):17–32. doi: 10.1007/s004120050278 . 9567198

[47] Kubota S, Takano J, Tsuneishi R, Kobayakawa S, Fujikawa N, Nabeyama M, et al. Highly repetitive DNA families restricted to germ cells in a Japanese hagfish (Eptatretus burgeri): a hierarchical and mosaic structure in eliminated chromosomes. Genetica. 2001;111(1–3):319–28. doi: 10.1023/a:1013751600787 . 11841177

[48] Baudry C, Malinsky S, Restituito M, Kapusta A, Rosa S, Meyer E, et al. PiggyMac, a domesticated piggyBac transposase involved in programmed genome rearrangements in the ciliate Paramecium tetraurelia. Genes Dev. 2009;23(21):2478–83. doi: 10.1101/gad.547309 19884254 PMC2779751

[49] Drotos KHI, Zagoskin MV, Kess T, Gregory TR, Wyngaard GA. Throwing away DNA: programmed downsizing in somatic nuclei. Trends Genet. 2022. Epub 20220225. doi: 10.1016/j.tig.2022.02.003 . 35227512

[50] Kloc M, Kubiak JZ, Ghobrial RM. Natural genetic engineering: A programmed chromosome/DNA elimination. Dev Biol. 2022. Epub 20220320. doi: 10.1016/j.ydbio.2022.03.008 . 35321809

[51] Borodin P, Chen A, Forstmeier W, Fouche S, Malinovskaya L, Pei Y, et al. Mendelian nightmares: the germline-restricted chromosome of songbirds. Chromosome Res. 2022. Epub 20220413. doi: 10.1007/s10577-022-09688-3 . 35416568 PMC9508068

[52] Rzeszutek I, Maurer-Alcala XX, Nowacki M. Programmed genome rearrangements in ciliates. Cell Mol Life Sci. 2020;77(22):4615–29. Epub 20200527. doi: 10.1007/s00018-020-03555-2 32462406 PMC7599177

[53] Muller F, Bernard V, Tobler H. Chromatin diminution in nematodes. Bioessays. 1996;18(2):133–8. Epub 1996/02/01. doi: 10.1002/bies.950180209 . 8851046

[54] Azzaria M, McGhee JD. DNA synthesis in the early embryo of the nematode Ascaris suum. Dev Biol. 1992;152(1):89–93. doi: 10.1016/0012-1606(92)90158-d . 1628758

[55] Gonzalez de la Rosa PM, Thomson M, Trivedi U, Tracey A, Tandonnet S, Blaxter M. A telomere-to-telomere assembly of Oscheius tipulae and the evolution of rhabditid nematode chromosomes. G3 (Bethesda). 2021;11(1). Epub 2021/02/10. doi: 10.1093/g3journal/jkaa020 33561231 PMC8022731

[56] Dockendorff TC, Estrem B, Reed J, Simmons JR, Zadegan SB, Zagoskin MV, et al. The nematode Oscheius tipulae as a genetic model for programmed DNA elimination. Curr Biol. 2022;32(23):5083–98 e6. Epub 20221114. doi: 10.1016/j.cub.2022.10.043 36379215 PMC9729473

[57] Rey C, Launay C, Wenger E, Delattre M. Programmed-DNA Elimination in the free-living nematodes *Mesorhabditis*. bioRxiv. 2022:2022.03.19.484980.

[58] Wang J. Genome Analysis of Programmed DNA Elimination in Parasitic Nematodes. Methods Mol Biol. 2021;2369:251–61. doi: 10.1007/978-1-0716-1681-9_14 34313993 PMC9173855

[59] Kisurina-Evgenieva OP, Sutiagina OI, Onishchenko GE. Biogenesis of Micronuclei. Biochemistry (Mosc). 2016;81(5):453–64. doi: 10.1134/S0006297916050035 . 27297896

[60] Niedermaier J, Moritz KB. Organization and dynamics of satellite and telomere DNAs in Ascaris: implications for formation and programmed breakdown of compound chromosomes. Chromosoma. 2000;109(7):439–52. Epub 2001/01/11. doi: 10.1007/s004120000104 . 11151673

[61] Muller F, Walker P, Aeby P, Neuhaus H, Felder H, Back E, et al. Nucleotide sequence of satellite DNA contained in the eliminated genome of Ascaris lumbricoides. Nucleic Acids Res. 1982;10(23):7493–510. doi: 10.1093/nar/10.23.7493 6296780 PMC327025

[62] Etter A, Aboutanos M, Tobler H, Muller F. Eliminated chromatin of Ascaris contains a gene that encodes a putative ribosomal protein. Proc Natl Acad Sci U S A. 1991;88(5):1593–6. Epub 1991/03/01. doi: 10.1073/pnas.88.5.1593 2000367 PMC51070

[63] Spicher A, Etter A, Bernard V, Tobler H, Muller F. Extremely stable transcripts may compensate for the elimination of the gene fert-1 from all Ascaris lumbricoides somatic cells. Dev Biol. 1994;164(1):72–86. Epub 1994/07/01. doi: 10.1006/dbio.1994.1181 . 8026638

[64] Huang YJ, Stoffel R, Tobler H, Mueller F. A newly formed telomere in Ascaris suum does not exert a telomere position effect on a nearby gene. Mol Cell Biol. 1996;16(1):130–4. Epub 1996/01/01. doi: 10.1128/MCB.16.1.130 8524289 PMC230986

[65] Jex AR, Liu S, Li B, Young ND, Hall RS, Li Y, et al. Ascaris suum draft genome. Nature. 2011;479(7374):529–33. Epub 2011/10/28. doi: 10.1038/nature10553 . 22031327

[66] Wang J, Czech B, Crunk A, Wallace A, Mitreva M, Hannon GJ, et al. Deep small RNA sequencing from the nematode Ascaris reveals conservation, functional diversification, and novel developmental profiles. Genome Res. 2011;21(9):1462–77. Epub 2011/06/21. doi: 10.1101/gr.121426.111 21685128 PMC3166831

[67] Moritz KB. Die Blastomerendifferenzierung fur Soma und Keimbahn bei Parascaris equorum. II. Untersuchungen mittels UV-Bestrahlung und Zentrifugierung. Wilhelm Roux’ Arch Entwicklungsmech. 1967;159:203–66. 10.1007/BF00573440 28304444

[68] Oliver BC, Shen SS. Cytoplasmic control of chromosome diminution in Ascaris suum. J Exp Zool. 1986;239:41–55. Epub 2005/05/11. doi: 10.1002/jez.1402390107

[69] Zagoskin MV, Wang J, Neff AT, Veronezi GMB, Davis RE. Small RNA pathways in the nematode Ascaris in the absence of piRNAs. Nat Commun. 2022;13(1):837. Epub 20220211. doi: 10.1038/s41467-022-28482-7 35149688 PMC8837657

[70] Cantara A, Luo Y, Dobrovolna M, Bohalova N, Fojta M, Verga D, et al. G-quadruplexes in helminth parasites. Nucleic Acids Res. 2022;50(5):2719–35. doi: 10.1093/nar/gkac129 35234933 PMC8934627

[71] Sanz LA, Hartono SR, Lim YW, Steyaert S, Rajpurkar A, Ginno PA, et al. Prevalent, Dynamic, and Conserved R-Loop Structures Associate with Specific Epigenomic Signatures in Mammals. Mol Cell. 2016;63(1):167–78. Epub 20160630. doi: 10.1016/j.molcel.2016.05.032 27373332 PMC4955522

[72] Morrison O, Thakur J. Molecular Complexes at Euchromatin, Heterochromatin and Centromeric Chromatin. Int J Mol Sci. 2021;22(13). Epub 20210628. doi: 10.3390/ijms22136922 34203193 PMC8268097

[73] Delamarre A, Barthe A, de la Roche Saint-Andre C, Luciano P, Forey R, Padioleau I, et al. MRX Increases Chromatin Accessibility at Stalled Replication Forks to Promote Nascent DNA Resection and Cohesin Loading. Mol Cell. 2020;77(2):395–410 e3. Epub 20191120. doi: 10.1016/j.molcel.2019.10.029 . 31759824

[74] Mehta A, Haber JE. Sources of DNA double-strand breaks and models of recombinational DNA repair. Cold Spring Harb Perspect Biol. 2014;6(9):a016428. Epub 20140807. doi: 10.1101/cshperspect.a016428 25104768 PMC4142968

[75] Dekker J, Misteli T. Long-Range Chromatin Interactions. Cold Spring Harb Perspect Biol. 2015;7(10):a019356. Epub 20151001. doi: 10.1101/cshperspect.a019356 26430217 PMC4588061

[76] Zheng H, Xie W. The role of 3D genome organization in development and cell differentiation. Nat Rev Mol Cell Biol. 2019;20(9):535–50. doi: 10.1038/s41580-019-0132-4 . 31197269

[77] Jerkovic I, Szabo Q, Bantignies F, Cavalli G. Higher-Order Chromosomal Structures Mediate Genome Function. J Mol Biol. 2020;432(3):676–81. Epub 20191102. doi: 10.1016/j.jmb.2019.10.014 . 31689436

[78] Muller F, Wicky C, Spicher A, Tobler H. New telomere formation after developmentally regulated chromosomal breakage during the process of chromatin diminution in Ascaris lumbricoides. Cell. 1991;67(4):815–22. Epub 1991/11/15. doi: 10.1016/0092-8674(91)90076-b 1934070

[79] Jentsch S, Tobler H, Muller F. New telomere formation during the process of chromatin diminution in Ascaris suum. Int J Dev Biol. 2002;46(1):143–8. Epub 2002/03/21. . 11902675

[80] Magnenat L, Tobler H, Muller F. Developmentally regulated telomerase activity is correlated with chromosomal healing during chromatin diminution in Ascaris suum. Mol Cell Biol. 1999;19(5):3457–65. Epub 1999/04/17. doi: 10.1128/MCB.19.5.3457 10207069 PMC84138

[81] Wang J, Garrey J, Davis RE. Transcription in pronuclei and one- to four-cell embryos drives early development in a nematode. Curr Biol. 2014;24(2):124–33. Epub 2014/01/01. doi: 10.1016/j.cub.2013.11.045 24374308 PMC3953457

[82] Canela A, Sridharan S, Sciascia N, Tubbs A, Meltzer P, Sleckman BP, et al. DNA Breaks and End Resection Measured Genome-wide by End Sequencing. Mol Cell. 2016;63(5):898–911. Epub 2016/08/02. doi: 10.1016/j.molcel.2016.06.034 27477910 PMC6299834

[83] Wong N, John S, Nussenzweig A, Canela A. END-seq: An Unbiased, High-Resolution, and Genome-Wide Approach to Map DNA Double-Strand Breaks and Resection in Human Cells. Methods Mol Biol. 2021;2153:9–31. Epub 2020/08/26. doi: 10.1007/978-1-0716-0644-5_2 . 32840769

[84] Goday C, Ciofi-Luzzatto A, Pimpinelli S. Centromere ultrastructure in germ-line chromosomes of Parascaris. Chromosoma. 1985;91(2):121–5. Epub 1985/01/01. doi: 10.1007/BF00294055 . 4039246

[85] Carlton PM, Davis RE, Ahmed S. Nematode chromosomes. Genetics. 2022. Epub 20220322. doi: 10.1093/genetics/iyac014 . 35323874 PMC9071541

[86] Perea-Resa C, Blower MD. Centromere Biology: Transcription Goes on Stage. Mol Cell Biol. 2018;38(18). Epub 20180828. doi: 10.1128/MCB.00263-18 29941491 PMC6113603

[87] Ekwall K. The roles of histone modifications and small RNA in centromere function. Chromosome Res. 2004;12(6):535–42. doi: 10.1023/B:CHRO.0000036584.40567.e5 . 15289661

[88] Gassmann R, Rechtsteiner A, Yuen KW, Muroyama A, Egelhofer T, Gaydos L, et al. An inverse relationship to germline transcription defines centromeric chromatin in C. elegans. Nature. 2012;484(7395):534–7. Epub 20120408. doi: 10.1038/nature10973 22495302 PMC3538161

[89] Krupina K, Goginashvili A, Cleveland DW. Causes and consequences of micronuclei. Curr Opin Cell Biol. 2021;70:91–9. Epub 20210218. doi: 10.1016/j.ceb.2021.01.004 33610905 PMC8119331

[90] Maciejowski J, Chatzipli A, Dananberg A, Chu K, Toufektchan E, Klimczak LJ, et al. APOBEC3-dependent kataegis and TREX1-driven chromothripsis during telomere crisis. Nat Genet. 2020;52(9):884–90. Epub 20200727. doi: 10.1038/s41588-020-0667-5 32719516 PMC7484228

[91] Tang S, Stokasimov E, Cui Y, Pellman D. Breakage of cytoplasmic chromosomes by pathological DNA base excision repair. Nature. 2022;606(7916):930–6. Epub 20220427. doi: 10.1038/s41586-022-04767-1 . 35477155 PMC10680091

[92] Dernburg AF, Sedat JW, Hawley RS. Direct evidence of a role for heterochromatin in meiotic chromosome segregation. Cell. 1996;86(1):135–46. doi: 10.1016/s0092-8674(00)80084-7 . 8689681

[93] Dernburg AF, McDonald K, Moulder G, Barstead R, Dresser M, Villeneuve AM. Meiotic recombination in C. elegans initiates by a conserved mechanism and is dispensable for homologous chromosome synapsis. Cell. 1998;94(3):387–98. doi: 10.1016/s0092-8674(00)81481-6 . 9708740

[94] MacQueen AJ, Phillips CM, Bhalla N, Weiser P, Villeneuve AM, Dernburg AF. Chromosome sites play dual roles to establish homologous synapsis during meiosis in C. elegans. Cell. 2005;123(6):1037–50. doi: 10.1016/j.cell.2005.09.034 16360034 PMC4435800

[95] Phillips CM, Dernburg AF. A family of zinc-finger proteins is required for chromosome-specific pairing and synapsis during meiosis in C. elegans. Dev Cell. 2006;11(6):817–29. doi: 10.1016/j.devcel.2006.09.020 . 17141157

[96] Cornes E, Bourdon L, Singh M, Mueller F, Quarato P, Wernersson E, et al. piRNAs initiate transcriptional silencing of spermatogenic genes during C. elegans germline development. Dev Cell. 2022;57(2):180–96 e7. Epub 20211217. doi: 10.1016/j.devcel.2021.11.025 34921763 PMC8796119

[97] Montgomery BE, Vijayasarathy T, Marks TN, Cialek CA, Reed KJ, Montgomery TA. Dual roles for piRNAs in promoting and preventing gene silencing in C. elegans. Cell Rep. 2021;37(10):110101. doi: 10.1016/j.celrep.2021.110101 34879267 PMC8730336

[98] Shin TH, Mello CC. Chromatin regulation during C. elegans germline development. Curr Opin Genet Dev. 2003;13(5):455–62. doi: 10.1016/s0959-437x(03)00109-6 . 14550409

[99] Etter A, Bernard V, Kenzelmann M, Tobler H, Muller F. Ribosomal heterogeneity from chromatin diminution in Ascaris lumbricoides. Science. 1994;265(5174):954–6. Epub 1994/08/12. doi: 10.1126/science.8052853 . 8052853

[100] Consortium CeS. Genome sequence of the nematode C. elegans: a platform for investigating biology. Science. 1998;282(5396):2012–8. doi: 10.1126/science.282.5396.2012 . 9851916

[101] Albertson DG, Nwaorgu OC, Sulston JE. Chromatin diminution and a chromosomal mechanism of sexual differentiation in Strongyloides papillosus. Chromosoma. 1979;75(1):75–87. Epub 1979/10/02. doi: 10.1007/BF00330626 . 533664

[102] Triantaphyllou AC, Moncol DJ. Cytology, reproduction, and sex determination of Strongyloides ransomi and S. papillosus. J Parasitol. 1977;63(6):961–73. . 592051

[103] Sulston JE, Brenner S. The DNA of Caenorhabditis elegans. Genetics. 1974;77(1):95–104. doi: 10.1093/genetics/77.1.95 4858229 PMC1213121

[104] Emmons SW, Klass MR, Hirsh D. Analysis of the constancy of DNA sequences during development and evolution of the nematode Caenorhabditis elegans. Proc Natl Acad Sci U S A. 1979;76(3):1333–7. doi: 10.1073/pnas.76.3.1333 286315 PMC383245

[105] Maddox PS, Oegema K, Desai A, Cheeseman IM. “Holo”er than thou: chromosome segregation and kinetochore function in C. elegans. Chromosome Res. 2004;12(6):641–53. doi: 10.1023/B:CHRO.0000036588.42225.2f . 15289669

[106] Melters DP, Paliulis LV, Korf IF, Chan SW. Holocentric chromosomes: convergent evolution, meiotic adaptations, and genomic analysis. Chromosome Res. 2012;20(5):579–93. doi: 10.1007/s10577-012-9292-1 . 22766638

